# The association between road traffic accidents and visual functions: A systematic review and meta-analysis

**DOI:** 10.4102/phcfm.v16i1.4601

**Published:** 2024-08-09

**Authors:** Gloria T. Tamenti, Tuwani A. Rasengane, Khathutshelo P. Mashige

**Affiliations:** 1 Department of Optometry, Faculty of Health Sciences, University of KwaZulu-Natal, Durban, South Africa; 2 Department of Optometry, Faculty of Health Sciences, University of the Free State, Bloemfontein, South Africa

**Keywords:** Vision function, road traffic accident, association, meta-analysis, systematic review

## Abstract

**Background:**

Poor visual functions have been reported to be related to the occurrence of road traffic accidents.

**Aim:**

To review the association between visual function and road traffic accidents (RTAs) from published relevant empirical studies.

**Setting:**

Worldwide.

**Methods:**

A random effects (RE) model meta-analysis was conducted using STATA 18. Statistical tests conducted include meta-summary statistics, RE meta-analysis (forest plot), meta-regression (relationship between mean age and effect sizes), funnel plots, Egger’s and Begg’s tests for publication bias and small study effects.

**Results:**

A total of 17 relevant studies, which were either cross-sectional or observational by design, were included in the meta-analysis. Reported effect sizes were within computed confidence intervals (CI) at 95%. The computed *Q* test of homogeneity was 61.94. The overall mean effect size of 1.43 (95% CI of 0.985–1.883) was statistically significant at a 5% level (*Z* = 6.26; *p* < 0.001). The *I*-squared = 62.17% (*p* = 0.00) confirmed moderate heterogeneity and the *Q*-value of 61.94 (*p* = 0.00) rejected the null hypothesis that the effect size was the same in all the studies. The funnel plot showed that the remaining majority of 13 studies were within the funnel plot on the right-hand side of the line of no effect.

**Conclusion:**

These results provide evidence of associations between visual functions and RTAs, and highlight the need for targeted interventions and further research to address the challenges associated with impaired visual functions and road safety.

**Contributions:**

The study contributes to the understanding of the implications of visual functions for road safety.

## Introduction

Road traffic accidents (RTAs) are one of the leading causes of death and disability worldwide, with an estimated 1.35 million fatalities and 50m injuries per year.^1^ The efforts to reduce their incidence and severity are a global public health priority. As with many public health systems, the burden of RTAs is higher in low- and middle-income countries.^2^ It is estimated that sub-Saharan Africa (SSA) has the highest number of RTA deaths in the world.^2^ South Africa is among the countries with the leading cause of death because of RTAs.^3^

Visual function (VF) plays a crucial role in driving safety. Visual acuity (VA), contrast sensitivity, visual fields and other aspects of VF are essential for detecting and responding to visual cues in the environment while driving.^4^ Impairments in VF, such as cataracts, macular degeneration and glaucoma, have been shown to increase the risk of RTAs.^5,6^ For example, Owsley et al.^6^ found that older adults with moderate or severe visual impairment had a 2.5 times greater risk of being involved in a motor vehicle crash than those without visual impairment.

Several studies have investigated the association between VF and RTAs. However, the findings have been inconsistent, and there is a lack of consensus on the strength and nature of the relationship between VF and RTAs. Some studies have found a significant association between VF and RTAs,^5,6,7^ while others have reported no association.^8,9^ Moreover, the studies have used different measures of VF, making it challenging to compare the results across studies.

To address these inconsistencies and provide a comprehensive synthesis of existing evidence, we conducted a meta-analysis of the association between VFs and RTAs. The objective of this study was to assess the strength of the association between VFs and RTAs, identify sources of heterogeneity and evaluate the quality of evidence.

## Methods

This systematic review was registered on the International Prospective Register of Systematic Reviews (PROSPERO registration number: CRD42023446292). The Preferred Reporting Items for Systematic Reviews and Meta-Analyses for Systematic Review Protocols (PRISMA-P) guidelines were used to develop and report the systematic review protocol. An electronic database online search was conducted on past and published empirical studies that assessed the association between VFs and RTAs, all published in English.

### Eligibility criteria

Past and published empirical studies included in this study satisfied the following criteria:

- Observational or experimental studies that assessed the association between VFs (VA or fields, contrast sensitivity, colour vision, depth) and RTAs.
- Studies conducted on human participants of each country’s legal driving age.
- Studies published or reported in the English language for easier access.

The following exclusion criteria were used:

- Studies that did not provide quantitative data on the association between VF and RTAs.
- Studies that included participants with neurological or cognitive impairments that could affect their ability to drive, such as patients with cortical/cerebral visual impairment.
- Studies that included paediatric patients who had not come of legal driving age.

No grey literature was included in this review.

### Search strategy

A comprehensive search strategy was developed in consultation with a research librarian. The following electronic databases were searched from January 1900 to 31 May 2023: MEDLINE, EMBASE, Cochrane Library and Web of Science. The search included keywords and medical subject headings (MeSH) related to VF, RTAs and driving. In addition, the study searched the reference lists of included studies and relevant reviews. These search terms ensured a comprehensive retrieval of relevant studies examining the association between RTAs and VF.

The search terms used were ‘Road traffic accidents’ AND ‘visual function’, ‘Car crashes’ AND ‘vision impairment’, ‘Traffic collisions’ AND ‘eye health’, ‘Motor vehicle accidents’ AND ‘visual acuity’, ‘Road safety’ AND ‘visual performance’, ‘Driving accidents’ AND ‘vision loss’, ‘Traffic incidents’ AND ‘ocular function’, ‘Vehicle collisions’ AND ‘sight impairment’, ‘Road accidents’ AND ‘visual defects’, ‘Driving crashes’ AND ‘eye disorders’, ‘Automobile accidents’ AND ‘vision tests’, ‘Road traffic injuries’ AND ‘visual problems’, ‘Traffic-related accidents’ AND ‘vision screening’, ‘Vehicle accidents’ AND ‘visual field’, ‘Traffic safety’ AND ‘visual impairment’, ‘Driving accidents’ AND ‘eye examination’, ‘Road traffic collisions’ AND ‘vision impairment’, ‘Crash risk’ AND ‘vision disorders’, ‘Road safety’ AND ‘visual acuity loss’, ‘Traffic accidents’ AND ‘eye disease’.

### Study selection

Two independent reviewers screened titles and abstracts of the identified studies for eligibility using the prespecified inclusion and exclusion criteria. Full texts of potentially eligible studies were then retrieved and assessed for eligibility. Discrepancies were resolved by discussion and consensus or by consulting a third reviewer.

### Data extraction

Two independent reviewers extracted data from the eligible studies using a predesigned data extraction form. The following information was extracted from each study: research study design, sample size, type of VF test used, exposure and outcome measures, effect size and confidence intervals (CIs), and other relevant information.

### Quality assessment

The risk of bias in the studies included was assessed independently by two reviewers using the Cochrane Risk of Bias tool for randomised controlled trials (RCTs) and the Newcastle-Ottawa Scale for observational studies. The quality of evidence was assessed using the Grading of Recommendations Assessment, Development and Evaluation (GRADE) approach based on the study design, risk of bias, inconsistency, indirectness, imprecision and publication bias. The quality of evidence was classified as high, moderate, low or very low. As the studies included were largely observational, this study used the Cochrane ROBINS-I tool to evaluate the quality of evidence in nonrandomised studies.^10^

### Data management and statistical methods

Data from eligible studies selected were compiled in MS Excel. The statistical tests conducted included meta-summary or descriptive statistics, random effects (RE) meta-analysis (forest plot), meta-regression (relationship between mean age and effect sizes), funnel plot, Egger’s and Begg’s tests for publication bias and small study effects.

### Descriptive analysis

Descriptive statistics were used to summarise the studies used, namely study author, country of study, proportions of RTA, mean age of participants and computed odds ratio (ORs).

### Meta-analysis

A meta-analysis was performed to estimate the pooled effect sizes and corresponding 95% CIs. A forest plot was generated to obtain the estimates of effect sizes and the overall pooled effect size using the RE model to account for the expected heterogeneity between studies. The primary outcome measure was the association between VF and RTAs. Visual functions covered in this study include impaired contrast sensitivity, visual impairments, VF defects (VA and contrast sensitivity), refractive error (presbyopia), glaucoma, severe visual defects (worse eye), severe contrast sensitivity impairment in both eyes, astigmatism, abnormal stereopsis and severe visual defect in the worse eye.

RTAs were defined as any motor vehicle collision (MVC) on any public road. To identify the potential source of heterogeneity based on the available data, a meta-regression testing the relationship between mean age (moderator variable) and meta-effect size was conducted. Stata statistical software version 18 for Windows was used to conduct all statistical tests and analyses covered in this study.

### Publication bias

Publication bias was assessed using funnel plots, Egger’s test and Begg’s test. Funnel plots were used to visualise the distribution of effect sizes relative to analogous standard errors. The Egger’s and Begg’s tests were used to assess symmetry and the extent of publication bias.

### Ethical considerations

Ethical clearance to conduct this study was obtained from the University of KwaZulu-Natal Biomedical Research Ethics Committee (BREC/00000664/2019).

## Results

**FIGURE 1:**
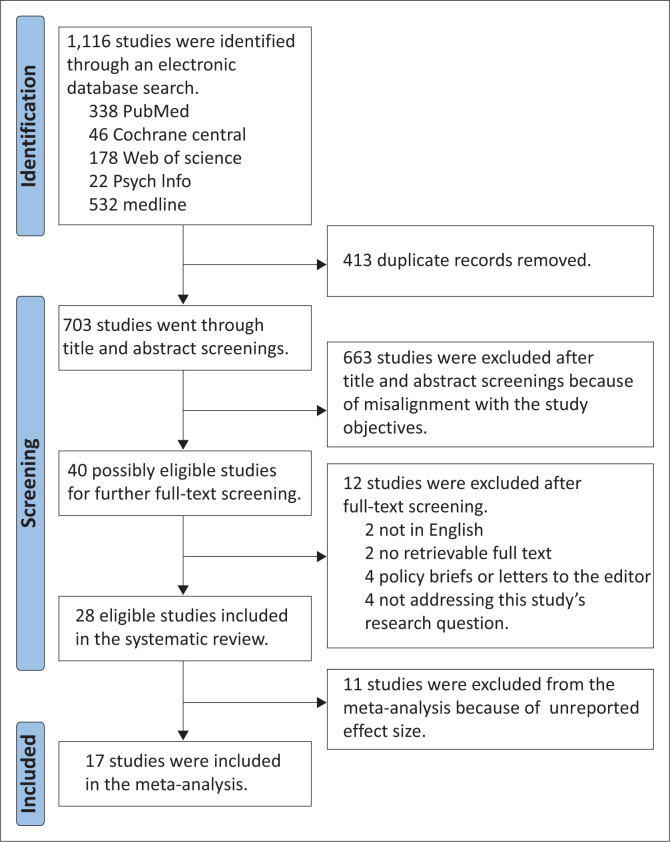
Preferred Reporting Items for Systematic Reviews and Meta-Analyses for Systematic Review Protocols flowchart of studies search.

The electronic database search yielded a total of 1116 studies, which were screened based on the search, and 413 duplicate studies were removed. The remaining 703 studies underwent further screening, of which 663 studies were excluded because they did not satisfy the research aim and objectives. The remaining 40 studies were subjected to full-text screening, and 12 of them were excluded for a variety of reasons, including the fact that they were not in English, did not have retrievable full text, were policy briefs or letters to the editor, or did not directly address the research question. Moreover, 28 studies were ultimately found to be eligible for meta-analysis. The studies were also the subject of a meta-analysis, and 11 of them were not included because ORs were either not reported or missing some aspects. A total of 17 (*n* = 17) studies were finally included in the meta-analysis (Figure 1). All studies included were either cross-sectional or observational by design – no interventional studies were identified.

Table 1 presents the summary statistics of studies on RTAs in different countries, including Bangladesh, Ghana, Nigeria, the United States, India, Japan, Iran and Birmingham. The studies sought to assess how RTAs are associated with various VFs such as VA, visual field, contrast sensitivity, colour vision, depth perception and eye diseases such as cataracts, glaucoma and refractive errors. The studies recruited diverse cohorts of drivers, including commercial (vehicle, bus and taxi) drivers and population drivers categorised by country, type of defect and type of driver.

**TABLE 1:** Descriptive analysis and characteristics of studies selection.

Author	Country	Total sample size	Mean age		Male		Female		Average number of years driving any vehicle	Cohort description	RTA (%)	Odds Ratio	Visual acuity	Contrast sensitivity		Colour		Depth	Cataract		Glaucoma		Refractive error		Visual field defects	
			*n*	%	*n*	%	*n*	%						*n*	%	*n*	%		*n*	%	*n*	%	*n*	%	*n*	%
Ahmed et al.,^11^ 2022	Bangladesh	700	42.3	10.0	700	100.0	0	-	16.1	Commercial bus drivers	9.0	2.45	18.0% (< 6/9)	NR	-	NR	-	NR	42	6.0	NR	-	492	70.0	NR	-
Boadi-Kusi et al.,^12^ 2015	Ghana	520	39.2	11.0	520	100.0	0	-	15.8	Commercial vehicle drivers	22.5	0.99	2.5% (< 6/9)	NR	-	32	6.2	NR	NR	-	NR	-	NR	-	NR	-
Ekpenyong et al.,^13^ 2020	Nigeria	2989	46.3	11.0	3157	90.0	351	10.0	NR	Population drivers	8.3	1.70	3.8% (< 6/18)	NR	-	NR	-	NR	NR	-	NR	-	171	49.0	NR	-
Huisingh et al.,^14^ 2014	United States of America	2000	74.5	-	1130	57.0	870	44.0	NR	Population drivers	14.0	1.83	8.1% (≤ 20/40)	132	7.0	NR	-	NR	NR	-	NR	-	NR	-	NR	-
Kumar et al.,^15^ 2022	India	382	34	-	382)	100.0	0	0.0	10	Commercial taxi drivers	29.6	2.6	8.1% (6/18)	NR	-	13	3.4	NR	NR	-	NR	-	312	60.0	NR	-
McGwin et al.,^16^ 2015	United States of America	438	72.8	-	195	44.0	243	55.0	NR	Population drivers	13.0	1.83	NR	NR	-	NR	-	NR	NR	-	438	100.0	NR	-	NR	-
Okamura et al.,^17^ 2019	Japan	546	59.8	-	438	80.0	108	20.0	NR	Population drivers	10.1	0.97	4.8% (< 6/18)	NR	-	NR	-	NR	NR	-	NR	-	11	8.0	NR	-
Oladehinde et al.,^18^ 2007	Nigeria	215	41.5	7.0	215	100.0	0	0.0	NR	Commercial vehicle drivers	2.8	3.50	3.3% (< 6/18)	NR	-	NR	-	NR	31	14.4	12	5.5	NR	-	NR	-
Owsley et al.,^19^ 2001	United States of America	377	75.5	-	197	52.0	180	42.0	NR	Population drivers	39.0	1.43	38.2% (< 20/50)	293	78.0	NR	-	NR	377	100.0	NR	-	NR	-	NR	-
Bekibele et al.,^20^ 2007	Nigeria	102	50.1	-	102)	100	0	-	27.94	Motor vehicle drivers	16.2	1.6	23.1% (< 6/9)	NR	-	NR	-	NR	NR	-	NR	-	NR	-	NR	-
Hashemi et al.,^21^ 2022	Iran	281	40.7	-	NR	-	NR	-	NR	Motor vehicle drivers	32.5	2.13	7.8% (≤ 20/40)	*p* ≤ 0.001	-	NR	-	NR	NR	-	NR	-	101	36.0	69	24.0
McGwin et al.,^22^ 2005	Birmingham, Alabama	120	72.5	-	68	57.0	52	43.0	NR	Motor vehicle drivers	70.0	4.40	NR	NR	-	NR	-	NR	106	87.0	NR	-	NR	-	NR	-
Owsley et al.,^6^ 1998	Alabama	294	38.2	-	156	54.0	138	47.0	NR	Motor vehicle drivers	22.8	2.20	12.6% (< 20/40)	58	20.0	NR	-	NR	NR	-	NR	-	NR	-	NR	-
Owsley et al.,^23^ 1998	Alabama	173	37.5	-	30	54.0	143	83.0	NR	Population drivers	10.3	5.78	NR	NR	-	NR	-	NR	NR	-	NR	-	NR	-	NR	-
Ovenseri-Ogomo and Adofo,^24^ 2011	Ghana	206	39.2	-	206	100.0	0	-	16.0	Commercial drivers	32.0	0.54	12.1 (< 6/9)	NR	-	7	3.4	NR	17	8.3	NR	-	66	32.0	14	7.0
Yuki et al.,^25^ 2014	Japan	247	62.1	-	172	70.0	75	30.0	NR	Population drivers	20.6	4.40	100% (< 6/12)	NR	-	NR	-	NR	NR	-	247	100.0	NR	-	NR	-

Note: Please see the full reference list of the article, Tamenti GT, Rasengane TA, Mashige KP. The association between road traffic accidents and visual functions: A systematic review and meta-analysis. *Afr J Prm Health Care Fam Med*. 2024;16(1), a4601. https://doi.org/10.4102/phcfm.v16i1.4601, for more information.

RTA, road traffic accident; NR, not reported.

The majority of studies comprised largely of male drivers, and the average age of the drivers covered in the sampled studies ranged between 34.0 and 75.5 years. The percentage of participants with a history of one or more RTAs ranged from 2.8% to 70.0%, and the ORs of the respective studies ranged from 0.54 to 5.78, which in essence converted into estimated effect sizes of the corresponding studies.

From the 17 studies included in the meta-analysis and assessed for methodological quality and risk of bias, effect sizes ranged from the lowest of 0.54 to the highest of 5.78, and the reported effect sizes were all within computed CI at 95% level. The computed Q test of homogeneity equal to 61.94 indicates the presence of moderate homogeneity across studies.

**FIGURE 2:**
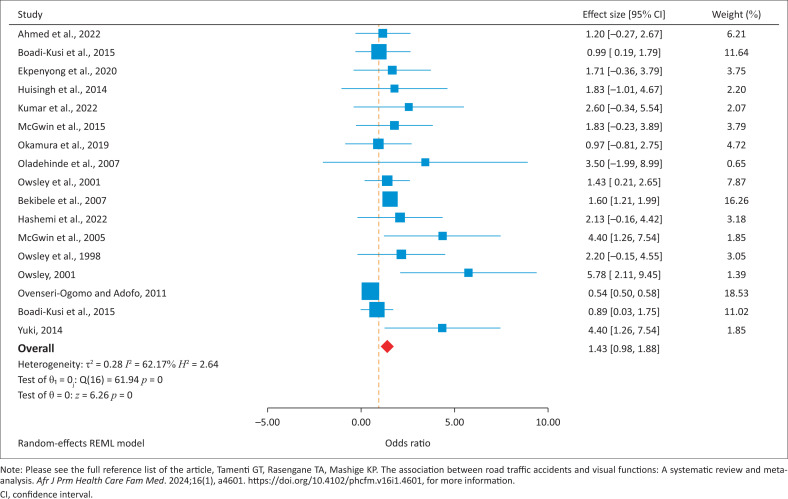
Forest plot of visual functions and road traffic accidents.

The overall mean effect size of 1.43 (95% CI of 0.98–1.88) statistically significant at a 1% level (*Z* = 6.26; *p* < 0.001) indicates that the average odds of someone with an abnormal VF being involved in a RTA were approximately 1.4 times higher than that of someone in the general population with normal VF. The *tau*-squared = 0.28 shows that the variance of the true effect was 0.28. These statistical results shown in Table 2 are visually shown in Figure 2. Figure 2 presents a forest plot of the RE meta-analysis – the main result of the study. The diagram visually illustrates the overall meta-analytic effect estimate (reported as OR) of the effect of VF on RTAs equal to 1.43 (95% CI of 0.98–1.88) statistically significant at 1% level (*Z* = 6.26; *p* < 0.001). In addition, results reveal an overall statistical heterogeneity of *I*^2^ = 62.2% (*p* < 0.01; *H*^2^ = 2.64; *Q* = 61.94), demonstrating moderate heterogeneity across the studies selected and used in this meta-analysis. A meta-regression was conducted, and the results showed that the average ages of drivers contributed to heterogeneity between and within sampled studies. There was no statistical evidence of heterogeneity as far as the rest of the characteristics were concerned.

**TABLE 2:** Meta-summary statistics (*N* = 17).

Study variable†	Effect size	95% CI	Weight (%)
Ahmed et al.^11^ 2022	1.200	−0.270, 2.670	6.210
Boadi-Kusi et al.^12^ 2015	0.990	0.186, 1.794	11.640
Ekpenyong et al.^13^ 2020	1.712	−0.361, 3.785	3.750
Huisingh et al.^14^ 2014	1.830	−1.012, 4.672	2.200
Kumar et al.^15^ 2022	2.600	−0.340, 5.540	2.070
McGwin et al.^16^ 2015	1.830	−0.228, 3.888	3.790
Okamura et al.^17^ 2019	0.970	−0.814, 2.754	4.720
Oladehinde et al.^18^ 2007	3.500	−1.988, 8.988	0.650
Owsley et al.^19^ 2001	1.430	0.215, 2.645	7.870
Bekibele et al.^20^ 2007	1.600	1.208, 1.992	16.260
Hashemi et al.^21^ 2022	2.130	−0.163, 4.423	3.180
McGwin et al.^22^ 2005	4.400	1.264, 7.536	1.850
Owsley et al.^6^ 1998	2.200	−0.152, 4.552	3.050
Owsley et al.^23^ 2001	5.780	2.115, 9.445	1.390
Ovenseri-Ogomo and Adofo,^24^ 2011	0.540	0.501, 0.579	18.530
Boadi-Kusi et al.^12^ 2015	0.890	0.028, 1.752	11.020
Yuki et al.^25^ 2014	4.400	1.264, 7.536	1.850
Theta	1.434	0.985, 1.883	-

Note: Test of theta = Ɵ: *z* = 6.26; Prob > ǀ *z* ǀ = 0.000; Test of homogeneity: Q = chi^2^(16) = 61.94; Prob > Q: 0.000; Effect size: Odds Ratio; Meta-analysis summary; Random-effects model; Method: REML; Heterogeneity: tau^2^ = 0.283; *I*^2^ (%) = 62.17; *H*^2^ = 2.64.

CI, confidence interval.

† , Please see the full reference list of the article, Tamenti GT, Rasengane TA, Mashige KP. The association between road traffic accidents and visual functions: A systematic review and meta-analysis. *Afr J Prm Health Care Fam Med*. 2024;16(1), a4601. https://doi.org/10.4102/phcfm.v16i1.4601, for more information.

**FIGURE 3:**
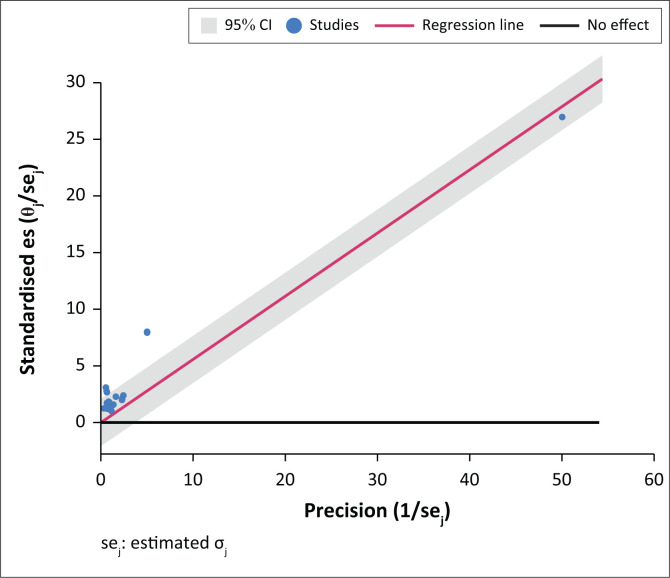
Heterogeneity test – Galbraith plot for summarising meta-analysis.

A further result confirming a moderate heterogeneity (distribution of what effect sizes are like) is the *I*-squared = 62.2% (*p* = 0.00), which assesses if the proportion of variance in observed effects is real. In other words, the reported I-squared indicates that 62.2% of the variance was the result of sampling error. The question of whether the true effect size could have been the same in all the research studies covered was addressed by the Q-value. Accordingly, the Q(18) value = 61.94 (*p* = 0.00) addressed the null hypothesis of no heterogeneity (Q(*df*), *p*-value). The reported *p* = 0.00 rejects the null hypothesis that the effect size is the same in all studies and concludes that the abnormal VFs have more of an effect in some populations than others (Figure 3).

Meta-regression results indicating the relationship between meta-effect sizes and mean age (in years) are reported in Table 3.

**TABLE 3:** Meta-regression of effect sizes and road traffic accidents (*N* = 16).

_meta_es	Coefficient	Std. err.	*z*-statistic	P > | *z* |	95% CI
Mean age	0.027	0.016	1.66	0.096	−0.004, 0.058
_cons	0.026	0.793	0.03	0.973	−1.527, 1.581

Note: Effect size: Odds ratio; random effects meta-regression; method: REML; residual heterogeneity; tau^2^ = 0.15; *I*^2^ (%) = 39.52; *H*^2^ = 1.65; *R*-squared (%) = 44.35; Wald chi^2^(1) = 2.76; Prob > chi^2^ = 0.096. Test of residual homogeneity: Q res = chi^2^(14) = 29.69 Prob > Q_res = 0.008.

_meta_es, meta effect size; Std. err., standard error; CI, confidence interval.

**FIGURE 4:**
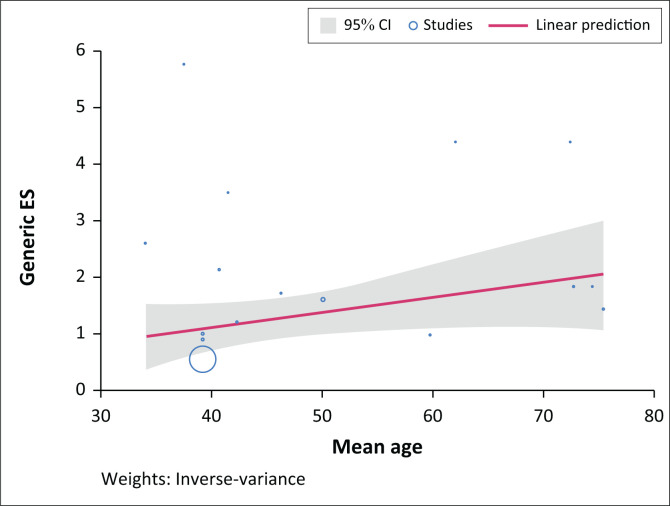
Bubble plot of the relationship between effect sizes and mean age.

Table 3 results indicate that a positive relationship exists between meta-effect sizes and the mean age of participants, despite being statistically insignificant at a 5% level. The tau-squared = 0.15 indicates that the variance of the true effect was 0.15 in terms of the influence of mean age on meta-effect sizes, while the reported I-squared indicates that 39.52% of the variance was the result of sampling error. Finally, the *R*-squared value indicates that 44.35% overall variation in meta-effect sizes was accounted for by the mean age of participants (Figure 4).

**FIGURE 5:**
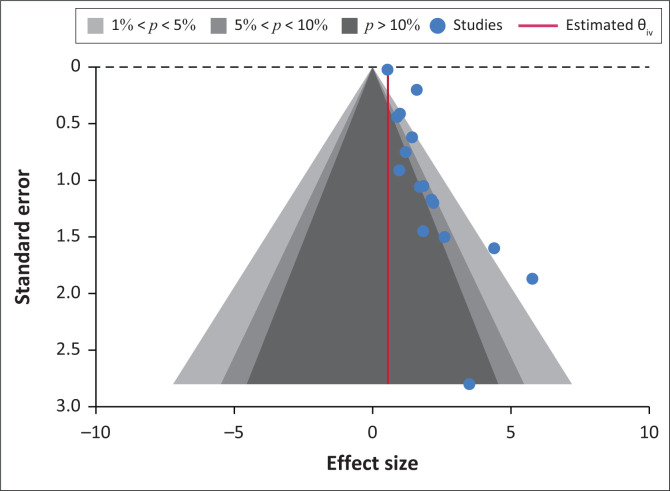
Funnel plot at 1%, 5% and 10% levels.

Figure 5 presents a funnel plot at 1%, 5% and 10% levels and Figure 5 assesses publication bias. A visual inspection revealed that the left-hand side (LHS) of the plot missed significant study results. Of the total 17 (*n* = 17) studies, only 3 did lie outside the funnel plot while the majority 16 did lie within the funnel plot on the right-hand side (RHS) of the line of no effect, indicating the presence of minimum publication bias across the studies used in this meta-analysis.

**FIGURE 6:**
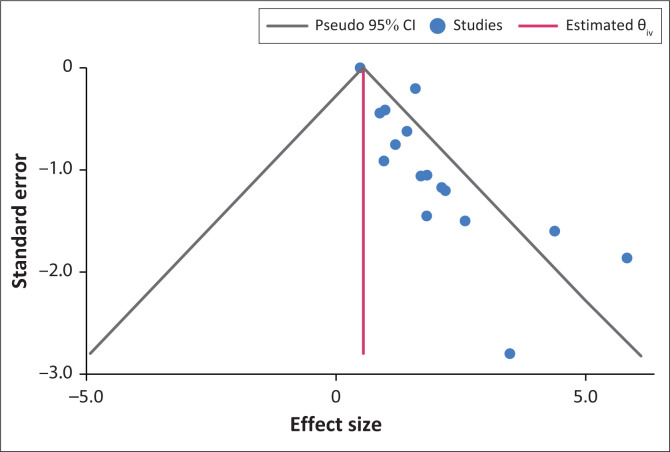
Funnel plot of publication bias.

Given the presence of minimum publication bias depicted in Figure 6, the possible presence of small study effects was tested using Egger’s test and Begg’s test (Table 4).

**TABLE 4:** Egger’s test and Begg’s test for small study effects.

Panel A: Egger’s test	Panel B: Begg’s test
Regression-based Egger’s test for small study	-
Random effects model	-
Method: REML	-
Moderators: mean age	-
**H0: beta1 = Ɵ; no small study effects**	
beta 1 = 1.30	Kendall’s score = 45.00
SE of beta1 = 0.433	SE of score = 28.235
*z* = 3.01	*z* = 1.82
Prob > ǀ *z* ǀ = 0.0026	Prob > ǀ *z* ǀ = 0.069

Note: Effect size: Odds ratio.

The statistically significant *p*-values at the 5% level suggest evidence of small study effects, thus confirming small publication bias. For correction of publication bias, the nonparametric trim-and-fill analysis was conducted, and results are presented in Table 5.

**TABLE 5:** Nonparametric trim-and-fill analysis of publication bias (*N* = 17).

Studies	Effect size	95% CI
Observed	1.434	0.985, 1.883
Observed + imputed	1.434	0.985, 1.883

Note: The trim-and-fill results for correction show no difference between the observed (OR = 1.434) and observed + imputed (OR = 1.434) values, hence there was significant publication bias. In other words, there was statistical evidence of a small study effect that is publication bias. Number of studies = 17 (observed = 17; imputed = 0). Iteration: Model: Random-effects; Method: REML. Pooling: Model: Random-effects; Method: REML.

CI, confidence interval.

## Discussion

This systematic review and meta-analysis aimed to investigate the relationship between VF and RTAs in distinct settings. From the 17 studies used in the systematic review and meta-analysis, only 2 (12%) provided evidence that exposure to VF defects was associated with lower odds of occurrence of RTAs (OR < 1). The studies include Boadi-Kusi et al.^12^ and Ovenseri-Ogomo and Adofo,^24^ with ORs of 0.9 and 0.5, respectively. Boadi-Kusi et al.^12^ tested the association between VFs (abnormal stereopsis, VA and colour vision defects) and RTA occurrence among commercial vehicle drivers in Ghana via cross-sectional design and multistage random sampling. Participants underwent a comprehensive eye examination after the administration of a structured questionnaire. With a mean VA of 0.02 ± 0.08 logMAR, results showed that there was no significant association between abnormal stereopsis (OR = 0.89; 95% CI: 0.44–1.80, *p* = 0.56) and occurrence of RTAs.

Ovenseri-Ogomo and Adofo^24^ determined the association between poor vision and the occurrence of RTAs among commercial drivers in the Cape Coast Municipality of Ghana using a cross-sectional design. Participants were subjected to an eye examination consisting of VA, colour vision testing using Ishihara pseudoisochromatic plates, confrontational visual fields, and external and internal ocular eye health examinations. A structured questionnaire was administered to participants to collect data on the history of driving and RTAs, utilisation of eye care services and identification of colours of traffic lights. Results revealed that the surveyed commercial drivers did not have the minimum VA required for driving, but there was no association between the occurrence of RTAs and visual fields (OR = 0.54).

Furthermore, the results of two studies by Boadi-Kusi et al.^12^ and Okamura et al.^17^ show that exposure to VFs among selected drivers did not affect their odds of being involved in RTAs (OR = 1). The ORs found by these respective studies regarding the association between VFs and RTA occurrences were all approximately equal to 1.0. Boadi-Kusi et al.^12^ found no association between myopia and RTA occurrence among commercial vehicle drivers in Ghana, whereas Okamura et al.^17^ found that ophthalmic indicators of binocular VF impairment like Esterman score and integrated visual fields did not significantly explain at-fault motor vehicle crash (MVC) involvement, both for predicting police-registered MVCs and self-reported at-fault MVC in Tokyo suburbs in Japan.

To a larger extent, the majority (13, 76%) of the studies covered in this systematic review and meta-analysis provided strong empirical evidence that exposure to VF defects was associated with higher odds of occurrence of RTAs (OR > 1). These studies include Ahmed et al.,^11^ Ekpenyong et al.,^13^ Huisingh et al.,^14^ Kumar et al.,^15^ McGwin et al.,^16^ Oladehinde et al.,^18^ Owsley et al.,^6,19^ Bekibele et al.,^20^ Hashemi et al.,^21^ McGwin et al.^22^ and Yuki et al.^25^ Findings from these studies indicating the significant association of VF defects and occurrence of RTAs are consistent with and/or confirm the overall meta-analytic effect estimate of the effect of VF defects on RTAs equal to 1.43 (95% CI of 0.98–1.88) statistically significant at 1% level (*Z* = 6.26; *p* < 0.001).

Ahmed et al.^11^ assessed the prevalence and causes of visual impairment among the bus drivers who underwent screening in Bangladesh and associations with self-reported crashes. Eye health screenings including refraction, and questionnaires were employed to collect data on near and distance visual impairment, and self-reported road traffic crashes among 700 drivers. The analysis revealed that 18% (*n* = 126) of the drivers presented VA in the better-seeing eye ≤ 6/9, which did not meet the vision standard of Bangladesh for bus drivers, and the majority 70% (*n* = 492) of drivers had near or distance refractive error. Further results showed that self-reported history of a motor vehicle crash (RTAs) was associated with near or distance visual impairment (OR = 2.5, 95% CI: 1.1–5.5), even after adjusting for other factors such as age and the distance driven. Bekibele et al.^20^ analysed the prevalence and risk factors of self-reported RTAs among drivers of educational institutions using a cross-sectional study design of motor vehicle drivers from the College of Medicine, University of Ibadan and University College Hospital Ibadan. Results indicate evidence of a strong significant association between RTA prevalence and visual impairment (OR = 1.6, 95% CI: 0.2–9.0).

Ekpenyong et al.^13^ determined the functional vision status of drivers in Nigeria and its association with occurrences of RTAs. Using a cross-sectional study design, vehicle drivers were interviewed, and clinical eye examinations were conducted by optometrists. The results from the analysis reveal that the drivers with visual impairment were nearly two times (adjusted OR = 1.7; 95% CI: 1.06–2.77) more likely to be involved in RTAs compared to those without visual impairment. Hashemi et al.^21^ assessed the relationship between visual field defects and RTA occurrences. Interviews and optometric and ophthalmic examinations were performed among participants. Results show evidence of a significant association between visual field defects in both eyes and RTA occurrences (adjusted OR = 2.13; 95% CI: 1.17–3.86).

Huisingh et al.^14^ designed a visual field test and assessed the associations between field impairment and MVC involvement among 2000 drivers aged 70 years and older. Results reveal that drivers with severe binocular field impairment in the overall driving visual field had about twice the odds of being involved in at-fault collision involvement (OR = 1.8; 95% CI: 1.07–1.83), concluding that older drivers with severe impairment in lower or left region of driving visual field are more likely to have a history of at-fault collision involvements.

Kumar et al.^15^ assessed the prevalence of refractive error and its association with RTAs and subsequent long-term spectacle compliance and adherence to suggested apt strategies in India. Logistic regression results indicate that drivers with refractive error were nearly two times (OR = 2.6; 95% CI: 1.4–5.1) more likely to be involved in RTAs compared to those without any refractive error, thereby confirming the significant association between refractive error (poor vision) and the occurrence of RTAs among drivers.

McGwin et al.^22^ examined the association between visual field defects in the central 24° field and the risk of MVCs among drivers with glaucoma. Results indicate that in the worse eye, drivers with severe field defects were at a significantly increased risk of MVC of about 4 times (OR = 4.4, 95% CI: 1.6–12.4) compared with those with no defects. Therefore, drivers with glaucoma and severe visual field impairment in the worse-functioning eye are at increased risk of involvement in a vehicle crash. McGwin et al.^22^ evaluated the association between binocular visual field defects among drivers with glaucoma and the risk of MVC involvement. Results show that drivers with severe visual fields were twice as likely to have an at-fault MVC compared to those not severely impaired (OR = 1.8, 95% CI: 0.81–2.74).

Oladehinde et al.^18^ determined the effects of the VFs on the occurrence of RTAs among commercial drivers in Nigeria using a cross-sectional study design and structured research instrument administered by ophthalmologists. Results showed a significant association between VA impairment in the better eye and RTA (OR = 3.5; 95% CI: 2.38–5.14). Owsley et al.^6^ tested whether measures of visual processing ability are associated with MVC occurrences by older drivers using a prospective cohort study with 3 years of follow-up during 1990–1993 via an ophthalmology clinic assessment of community-based setting among a sample of 294 drivers aged 55–87 years at enrolment. Results indicate that older drivers with a 40% or greater impairment in the useful field of view were 2.2 times (OR = 2.2; 95% CI: 1.2–4.1) more likely to be involved in MVC during 3 years of follow-up, after adjusting for age, sex, race, chronic medical conditions, mental status and days driven per week.

Owsley et al.^23^ examined the association between severe contrast sensitivity impairment and the occurrence of increased motor vehicle crash risk of older drivers with cataracts using a cross-sectional research design. The dependent variable was involvement in at least one state-recorded, at-fault vehicle crash during 5 years before study enrolment. Logistic regression results indicated that drivers who had severe contrast sensitivity impairment in both eyes were 6 times more likely (OR = 5.78; 95% CI: 1.87–17.86) involved in RTAs than crash-free drivers.

Moreover, the odds of drivers who had VA in the range of 20/25–20/30 were 1.43 times higher than those whose VA was 20/25 or better. Yuki et al.^25^ investigated the relationship between visual field defect and history of MVCs in subjects with primary open-angle glaucoma (POAG). Drivers’ driving attitudes were estimated using Rasch analysis and results revealed a significant association (OR 4.4, 95% CI: 1.6–12.4) between moderate VF defects and the occurrences of RTAs among drivers.

Although the majority of studies provide strong evidence of significant associations between VFs and RTA occurrences, many conditions contributed to heterogeneity within and between studies. For example, depth perception and contrast sensitivity influence the driver’s ability to accurately judge the distances, speed and appearance of obstacles on roads. Most studies did not explore these variables in detail, while among those that did so, sample sizes were disproportionate, and regions and populations were different, which could potentially explain the heterogeneity. Notwithstanding the methodological differences, the implications of these conditions and other confounders on RTA occurrences have been studied and the results of meta-analysis reported herein are consistent with previous studies that have shown a significant relationship between VFs and RTA occurrences.

In addition, studies featured diverse driver cohorts, ranging from commercial vehicle drivers to general population drivers. Notably, a gender bias was considerable, with most participants being male. As the prevalence of RTAs varies across different nations, socioeconomic factors influencing road safety also need to be explored in that regard. For instance, highly developed countries may have advanced infrastructure, stringent regulations and better enforcement mechanisms, thus contributing to lower accident rates. In contrast, developing nations might struggle with challenges such as inadequate and poor road infrastructure, lax enforcement and limited access to healthcare, potentially exacerbating the impact of VF factors on road safety.^26^ Understanding these peculiarities is essential for tailoring target interventions that address specific needs and challenges faced by different groups of drivers with different states of VFs. The findings of this systematic review provide valuable insights into the association between RTAs and VF. The identified small study effects and publication bias necessitate a careful and nuanced interpretation of the results. Addressing these biases in future research will enhance the reliability and applicability of the evidence in this field.

## Conclusion

The major results (overall meta-analytic effect) of this systematic review and meta-analysis elucidate the relationship between VF defects and the occurrences of RTAs. The broad synthesis of studies from diverse countries has a broad spectrum of prevalence rates for visual impairments, revealing the multifaceted nature of impairments within the driving population. The meta-analysis reveals a significant overall effect, indicating that individuals with VF defects have higher odds of being involved in RTAs compared to those with normal VFs. Therefore, relevant road traffic legislation and policy measures should be developed and implemented to reduce the odds of RTA occurrences. In addition, the government and responsible transport sector authorities should also implement targeted interventions aimed at curbing and eradicating RTAs caused by human negligence particularly when the drivers at fault knew that their visual conditions were not good for driving.

Visual field defects are a significant risk factor that influences the prevalence of RTAs; hence, special attention and necessary interventions should be given to individuals with such type of a defect. Ensuring that drivers of all categories (commercial, noncommercial and general population) are screened to meet the required visual standards for driving while referring those who do not for treatment can be considered to contribute towards safer roads in high-risk settings. Therefore, regular examination of drivers’ eyes should be strongly recommended and practised regularly at consistently reasonable time intervals.
